# Wet oxidation and catalytic wet oxidation of pharmaceutical sludge

**DOI:** 10.1038/s41598-022-22847-0

**Published:** 2023-02-13

**Authors:** Xu Zeng, Jun Liu, Jianfu Zhao

**Affiliations:** 1 State Key Laboratory of Pollution Control and Resources Reuse, College of Environmental Science and Engineering, Tongji University, 1239 Siping Road, Shanghai, 200092 China; 2 Shanghai Electric Power Generation Environment Protection Engineering Co., Ltd., Shanghai, 201612 China

**Keywords:** Pollution remediation, Pollution remediation

## Abstract

In this work, wet oxidation and catalytic wet oxidation of pharmaceutical sludge using homogeneous and heterogeneous catalysts were investigated. The results indicate that wet oxidation is a promising method for the highly efficient degradation of pharmaceutical sludge. Under optimal conditions, the highest removal efficiencies of volatile suspended solids (VSS) 86.8% and chemical oxygen demand (COD) 62.5% were achieved at 260 °C for 60 min with an initial oxygen pressure of 1.0 MPa. NaOH exhibited excellent acceleration performance on the VSS removal. The highest VSS removal efficiency of 95.2% was obtained at 260 °C for 60 min with an initial oxygen pressure of 1.0 MPa and 10 g·L^−1^ of NaOH. By using a Cu–Ce/γ-Al_2_O_3_ catalyst, the highest removal rates of VSS 87.3% and COD 72.6% were achieved at 260 °C for 60 min with an initial oxygen pressure of 1.0 MPa and 10 g·L^−1^ of catalyst. The wet oxidation reaction can be maintained itself owing to the exothermic heat. The produced low-molecular-weight carboxylic acids have potential commercial utilization as organic carbon sources in the biological wastewater treatment processes. The inorganic residues can be utilized for the building materials production. These results implied that the catalytic wet oxidation is a promising method for the volume reduction and resource utilization of pharmaceutical sludge.

## Introduction

Large amounts of pharmaceutical sludge are generated from pharmaceutical industries, posing a high environmental risk due to the hazardous and refractory organic pollutants contained in the sludge^1^. Pharmaceutical sludge is a complex and hazardous industrial waste because it contains a variety of toxic compounds. In particular, pharmaceutical sludge contains relatively high levels of soluble organics, heavy metals, and recalcitrant antibiotics, such as benzylpenicillin, aureomycin, and berberine hydrochloride that should be treated as hazardous wastes^2–4^. The treatment of pharmaceutical sludge has attracted increasing interest. Traditionally, biotechnology and incineration have been applied for the treatment of pharmaceutical sludge. However, toxic pollutants are lethal to microorganisms in bioprocesses with low efficiency. The incineration process merely transfers the pollutants from a liquid to a solid or to air, releasing noxious compounds (oxides of sulphur and nitrogen, furan, etc.) into the air. In addition, the sludge disposal process is complicated and costly^5^. Therefore, an effective, economical and green process for the treatment of pharmaceutical sludge is strongly desired.

Wet oxidation (WO) is one of the numerous technologies that have been studied for sludge minimization and treatment^6^. In this process, organic pollutants are converted to low-molecular-weight carboxylic acids, carbon dioxide, and water and inorganic salts without the generation of harmful emissions. The low-molecular-weight carboxylic acid intermediates can be used to promote anaerobic fermentation^7^. Therefore, wet oxidation is an interesting alternative for the mineralization of activated sludge^8–10^. Gasso et al. reported the use of a compact jet-mixer reactor as a promising WO strategy^11^. However, to obtain reasonably high removal and conversion rates, the wet oxidation reaction requires high temperature and pressure^12^. Over the past decades, extensive studies on catalytic wet air oxidation (CWAO) have been conducted focusing on the development of catalysts^13,14^. The addition of catalysts can decrease the operating temperature, enhance the reaction rate, and shorten the residence times^15,16^. Among various catalysts, catalysts containing Cu^2+^ or Ce^3+^ have attracted considerable attention due to their good catalytic results for the oxidation of wastewater and sludge. Wang et al. studied the wet air oxidation of pharmaceutical wastewater by a Cu^2+^ system^17^. It was shown that the biodegradability of poisonous, harmful, and hard-to-degrade organic wastewater can be greatly improved^18^. Cu^2+^ can also act as efficient catalysts^19^. Zhang et al. reported that a Cu–Ni bimetallic-based catalyst synthesized under microwave irradiation with a two-dimensional macrostructure enhanced the catalytic effect^20^. It was reported that NaOH can be used in the oxidation of alcohols as a homogeneous catalyst^21^. Several studies on the CWO of organic compounds also proved the catalytic effects of NaOH^22,23^. OH^−^ is considered to act as an initiator or promoter that abstracts a proton from the hydroxyl group. The presence of NaOH affects not only the reaction efficiency but also the selectivity of the oxidation reactions. However, only a few studies have been conducted using real industrial sludge. Therefore, it is important to examine the treatment of real industrial pharmaceutical sludge using wet oxidation method.

In this study, wet oxidation and catalytic wet oxidation of pharmaceutical sludge with several different catalysts were investigated. The removal efficiency of VSS and COD were evaluated.

## Material and methods

### Characteristics of the pharmaceutical sludge

The pharmaceutical sludge was collected from a synthetic pharmaceutical factory located in eastern China. The sludge was extracted from a thickening unit and stored at 4 °C until further usage. The characteristics of the raw sludge are as follows: COD 15,000–16,000 mg/L, VSS 13.5–13.8 g/L, VSS/SS ratio 39–40%, pH 7.5–8.0. The sludge was used in the experiments without any treatment. Experimental reagents, such as Cu(NO_3_)_2_, Ce(NO_3_)_3_, Ni(NO_3_)_2_, Fe_2_(SO_4_)_3_, and NaOH, were purchased from Shanghai Sinopharm Chemical Reagent Co., Ltd. All of the reagents used in this study were of analytical grade and were used as received without further purification. The gaseous oxygen used as the oxidant was commercial industrial gas.

### Experimental system

**Figure 1:**
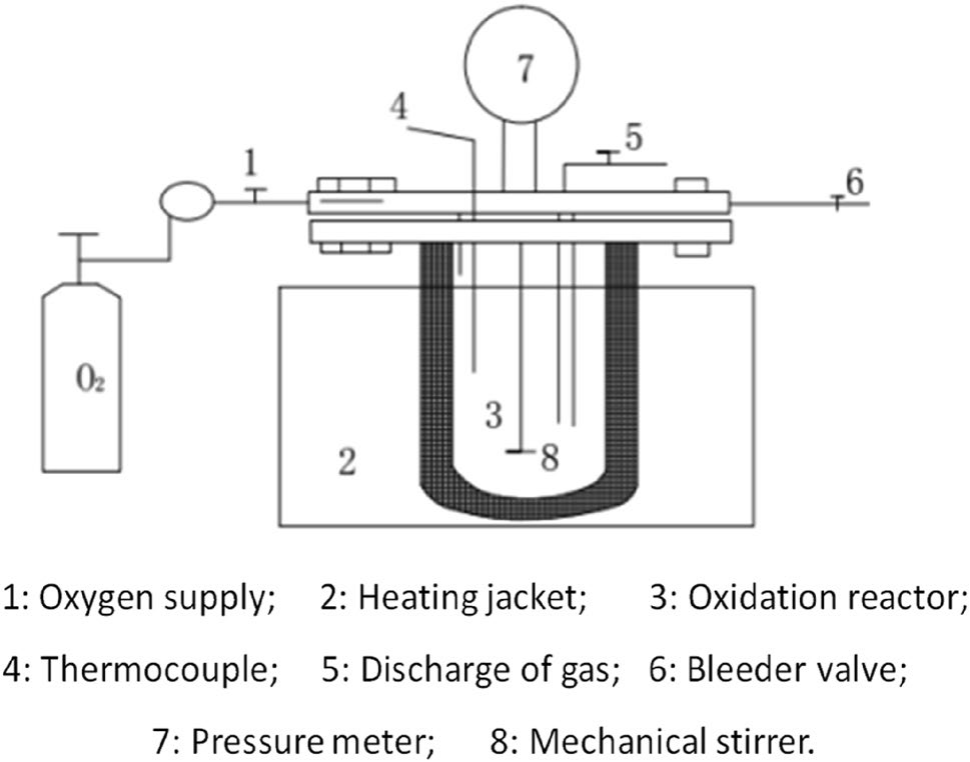
Diagram of wet oxidation reactor.

All experiments in this study were conducted in a 100 mL SUS316 autoclave reactor equipped with a mechanically driven stirrer, as shown in Fig. 1. The reactor was purchased from Anhui Kemi Machinery Technology Co. Ltd, China^24^. The temperature was varied in the range of 180–260 °C using an electric jacket, and the initial oxygen partial pressure was varied in the range of 0.2–1.0 MPa. The stirring speed was 300 rpm. The typical experimental procedure was as follows: pharmaceutical sludge solution (50 mL) and a certain amount of catalyst, if necessary, were placed into the reactor, and then the reactor was closed. The oxygen gas was added to the reactor at the beginning. The oxygen gas was injected with desired pressure after several times purge to replace the nitrogen gas in the reactor. After that, the reactor was heated to the expected temperature. The pressure in the reactor was due to self-pressurization with saturated vapour pressure. Once the desired temperature was reached, this moment was taken as the zero time of the reaction. After the desired reaction time, the reactor was removed from the oven and allowed to cool to room temperature. After the liquid was cooled to room temperature, it was sampled and analyzed.

### Analytical method

VSS was measured by the ignition loss method, which was performed at 550 ± 50 °C for 4 h, after drying at 105 °C for 2 h. The samples were weight after they are cooled in a dessicator. COD was measured by the potassium dichromate oxidation method (Hach Heating System, Hach Corporation, USA). pH was measured with a pH metre (pH-201, Hanna Corporation, Italy). The surface morphologies of the prepared catalysts and the carrier were examined by scanning electron microscopy (Agilent 8500 FE-SEM). The elemental content of the catalysts was analysed by X-ray diffraction (XRD-7000, Japan) using a Siemens model with Cu-Kα radiation.

## Results and discussion

### Wet oxidation of pharmaceutical sludge

**Figure 2:**
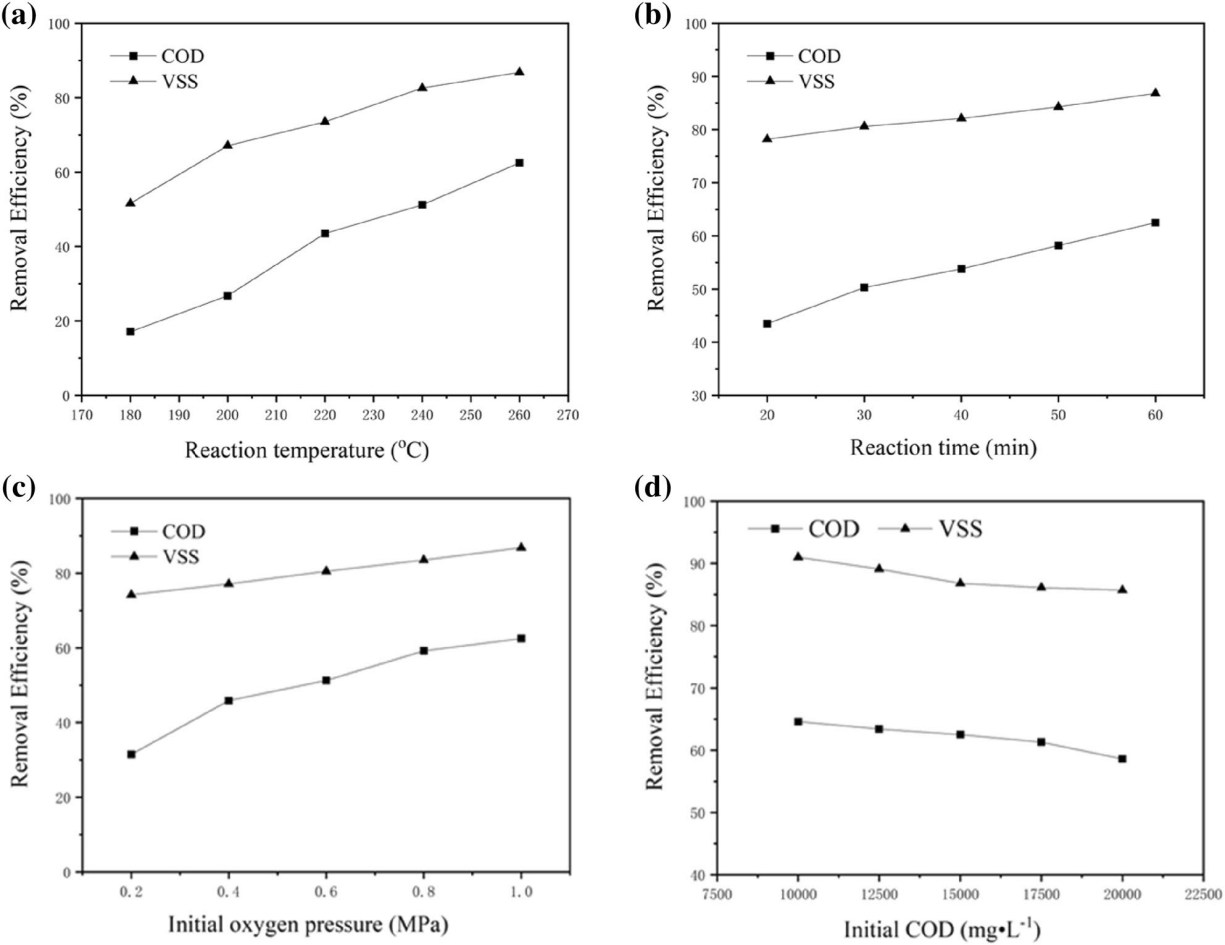
Effect of reaction conditions on the VSS and COD removal efficiencies.

Several reaction parameters that may affect the removal efficiency, including the reaction temperature and time, initial oxygen pressure and initial COD, were investigated. Under optimal conditions, the highest removal efficiency of VSS 86.8% was achieved at 260 °C for 60 min with an initial oxygen pressure of 1.0 MPa and initial COD 15,000 mg·L^−1^. At the same time, the removal efficiency of COD reached as high as 62.5%. As shown in Fig. 2a, the effect of the reaction temperature is quite significant. The COD and VSS removal rates increased with increasing temperature. When the temperature increased from 240 to 260 °C, the increase in the VSS removal efficiency was not strong. This is most likely because the thermal hydrolysis process was almost finished even at 240 °C. However, the COD removal rate was not high. This may be because the products of the wet oxidation of organics are mainly low-molecular-weight carboxylic acids such as formic acid or acetic acid that are not easily oxidized under hydrothermal conditions. Therefore, incomplete degradation of COD occurred. We conclude that higher temperatures were favourable for the wet oxidation of pharmaceutical sludge. However, from a practical point of view, higher temperatures lead to higher operating costs. As shown in Fig. 2b, with increasing time, the removal rates gradually increased. Because thermal hydrolysis can occur easily within a short time, the removal efficiency of VSS was much higher than that of COD with the same reaction time. Oxygen played an important role in VSS and COD removal. Figure 2c shows that COD removal increases with increasing oxygen pressure. This behaviour was expected because the increase in the oxidant concentration usually leads to an increase in the oxidation rate. These results indicate that high oxygen pressures effectively eliminate refractory and toxic organic compounds. A high initial COD means a high concentration of pollutants in the sludge solution; therefore, the VSS and COD removal efficiencies decreased as the initial COD increased (see Fig. 2d). However, the efficiency of the wet oxidation of the total pollutant amount decreased. If the initial COD was too low, the exothermic reaction could not be maintained. These results imply that wet oxidation is a promising method for highly efficient degradation of pharmaceutical sludge.

### Effects of metal salts on the removal efficiency

**Figure 3:**
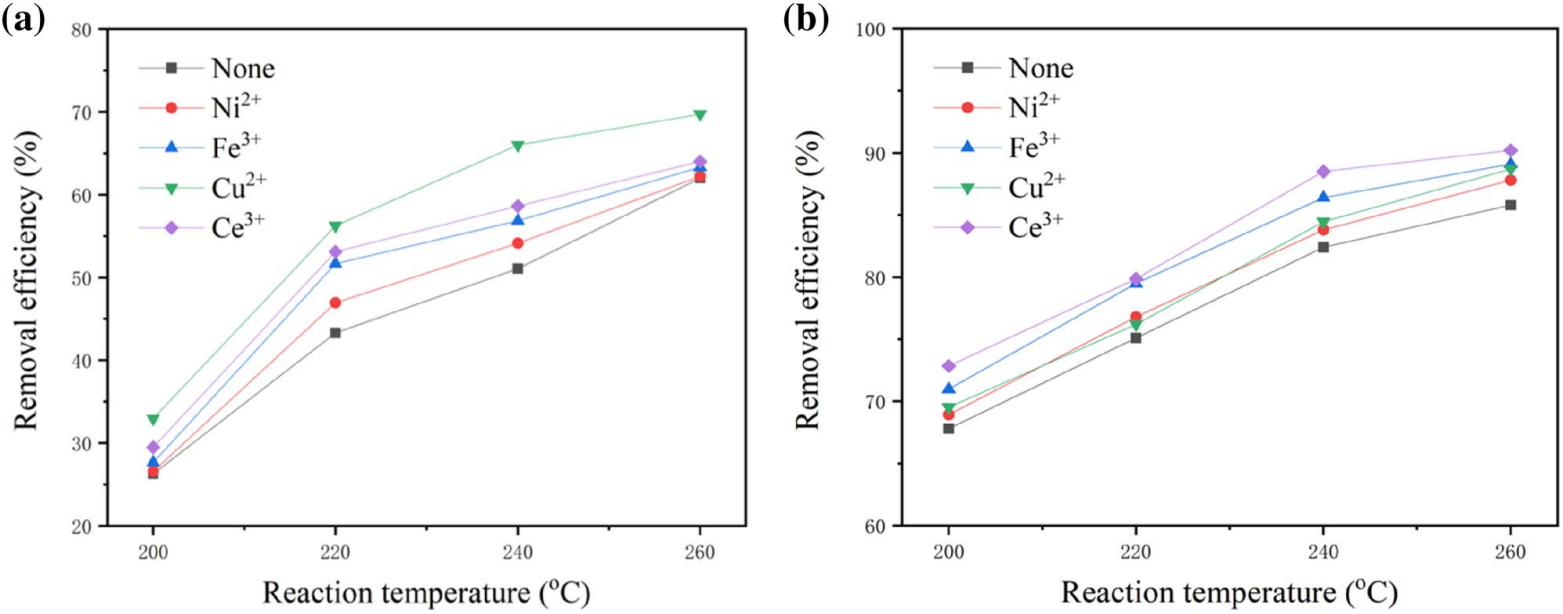
Effect of metal ions on the removal efficiency of COD (**a**) and VSS (**b**).

Metal salts were chosen as homogeneous catalysts to examine their effects on the wet oxidation of pharmaceutical sludge, including Cu(NO_3_)_2_, Ce(NO_3_)_3_, Ni(NO_3_)_2_, Fe_2_(SO_4_)_3_. Experiments were performed at 200, 220, 240 and 260 °C separately with a reaction time of 60 min, an initial oxygen supply of 1.0 MPa and 0.2 g·L^−1^ of catalyst. As shown in Fig. 3a, the reaction temperature played an important role in the catalytic wet oxidation process. In particular, when the temperature increased from 200 to 220 °C, the removal efficiency of COD increased by more than 25%. Compared with the results in the absence of catalyst, the removal efficiencies of COD with homogeneous catalysts increased up to above 10%. These results illustrated that the homogeneous catalysts chosen in this study can accelerate the wet oxidation of pharmaceutical sludge. By comparing the results with different catalysts, the promotional effect of Cu^2+^ is the highest. The highest removal efficiency of COD, approximately 70%, was obtained with Cu^2+^ at 260 °C. As shown in Fig. 3b, compared with the removal efficiencies of COD, the efficiencies of VSS increased only slightly, typically in the approximately 3–5% range. The effects of Cu^2+^ and Ce^3+^ were significant. As the temperature increased from 200 to 260 °C, the removal efficiencies of VSS increased from approximately 70% to 90%. Even at a reaction temperature of 200 °C, the removal efficiency of VSS was approximately 70%, which means that thermal hydrolysis can occur easily compared to the COD results. The highest removal efficiency of VSS was almost 90%; however, it was only 70% for COD. This may be because thermal hydrolysis induced the solubilization of the sludge, and the wet oxidation of the pollutants in the liquid may produce acetic acid and other low-molecular-weight carboxylic acids that are not easily oxidized.

### Effects of NaOH on the removal efficiency

**Figure 4:**
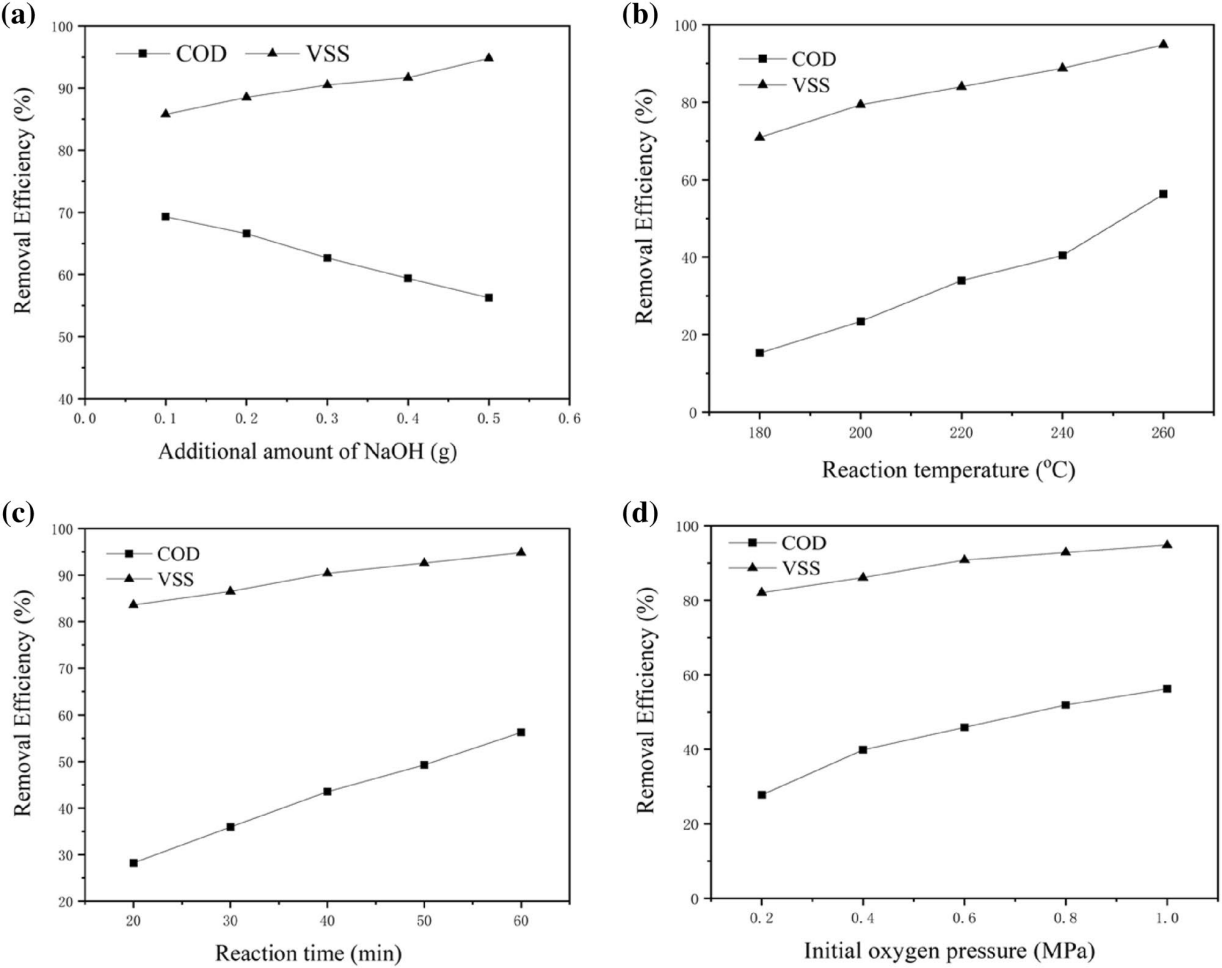
Effect of NaOH on the VSS and COD removal efficiencies.

Several studies on the catalytic wet oxidation of organic compounds proved the catalytic effects of NaOH as a homogeneous catalyst. OH^−^ is considered to act as an initiator or promoter that abstracts a proton from the hydroxyl group. The presence of NaOH affects not only the reaction efficiency but also the selectivity of the oxidation reactions^22^. Therefore, experiments were conducted to investigate the effects of NaOH. The effects of the additional amount of NaOH, reaction temperature and time, and initial oxygen supply were examined, as shown in Fig. 4a –d. It is observed from Fig. 4a that the VSS removal efficiencies increased significantly with increasing amount of the added NaOH. The maximum value of VSS removal (95.2%) was obtained in the presence of 10.0 g·L^–1^ NaOH. By contrast, the COD removal rate reached 57.3% in this case. Interestingly, the COD removal rate decreased with the addition of NaOH. This may be because the addition of NaOH induced the accumulation of acetic acid which is a refractory compound^25^. Considering that the carboxylic acids produced in the wet oxidation process can be easily disposed of in biological wastewater treatment, the liquid after the wet oxidation process can be used to increase the BOD/COD ratio of the wastewater. Comparison to the results with metal ions shown in Fig. 3 reveals that the enhancement effect of NaOH for the VSS removal was higher, even though the metal ions are more effective as catalysts for the removal of COD. These results showed that the addition of NaOH enhanced the hydrolysis of the sludge significantly.

### Preparation of Cu–Ce/γ-Al_2_O_3_ catalyst

**Figure 5:**
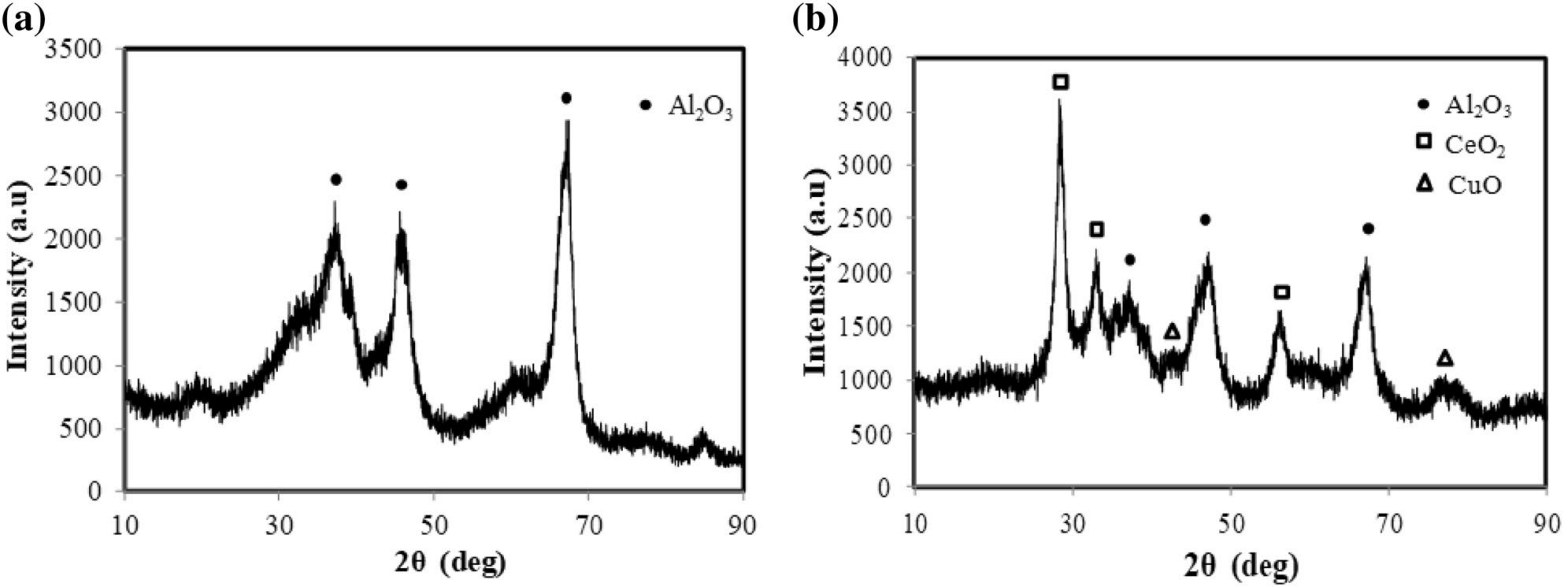
Characterization results of the prepared catalyst.

Among various catalysts in the catalytic wet oxidation process, catalysts containing Cu^2+^ or Ce^4+^ have attracted considerable attention due to their good performance in the oxidation of wastewater and sludge. Copper species are less pH-dependent and have received considerable research attention in the last ten years because the redox reaction with Cu species is more stable^26,27^. In our previous study, we also investigated several catalysts for the wet oxidation of pharmaceutical sludge and wastewater. We found that the prepared Cu–Ce/γ-Al_2_O_3_ catalyst has good performance^28–30^. The catalyst was synthesized by a typical wet impregnation procedure. First, Cu(NO_3_)_2_ and Ce(NO_3_)_3_ were mixed in the 1:1 molar ratio at the concentration of 1.0 mol/L. Then, γ-Al_2_O_3_ acting as the substrate was placed in the liquor for 24 h. After impregnation, the prepared solids were washed with deionized water for three times, then it was dried at 120 °C overnight and calcined at 550 °C for 4 h. The characterization results of the prepared catalyst are shown in Fig. 5. The XRD patterns of the solid products before and after loading are shown in Fig. 5a,b. It is quite clear that new peaks were detected after loading. Prior to loading, the XRD pattern showed only the peaks for γ-Al_2_O_3_. After loading, new peaks from the loaded CuO–CeO_2_ were clearly visible. Then, catalytic wet oxidation of the pharmaceutical sludge was performed using the synthesized catalyst.

### Effect of the Cu–Ce/γ-Al_2_O_3_ catalyst on the removal efficiency

**Figure 6:**
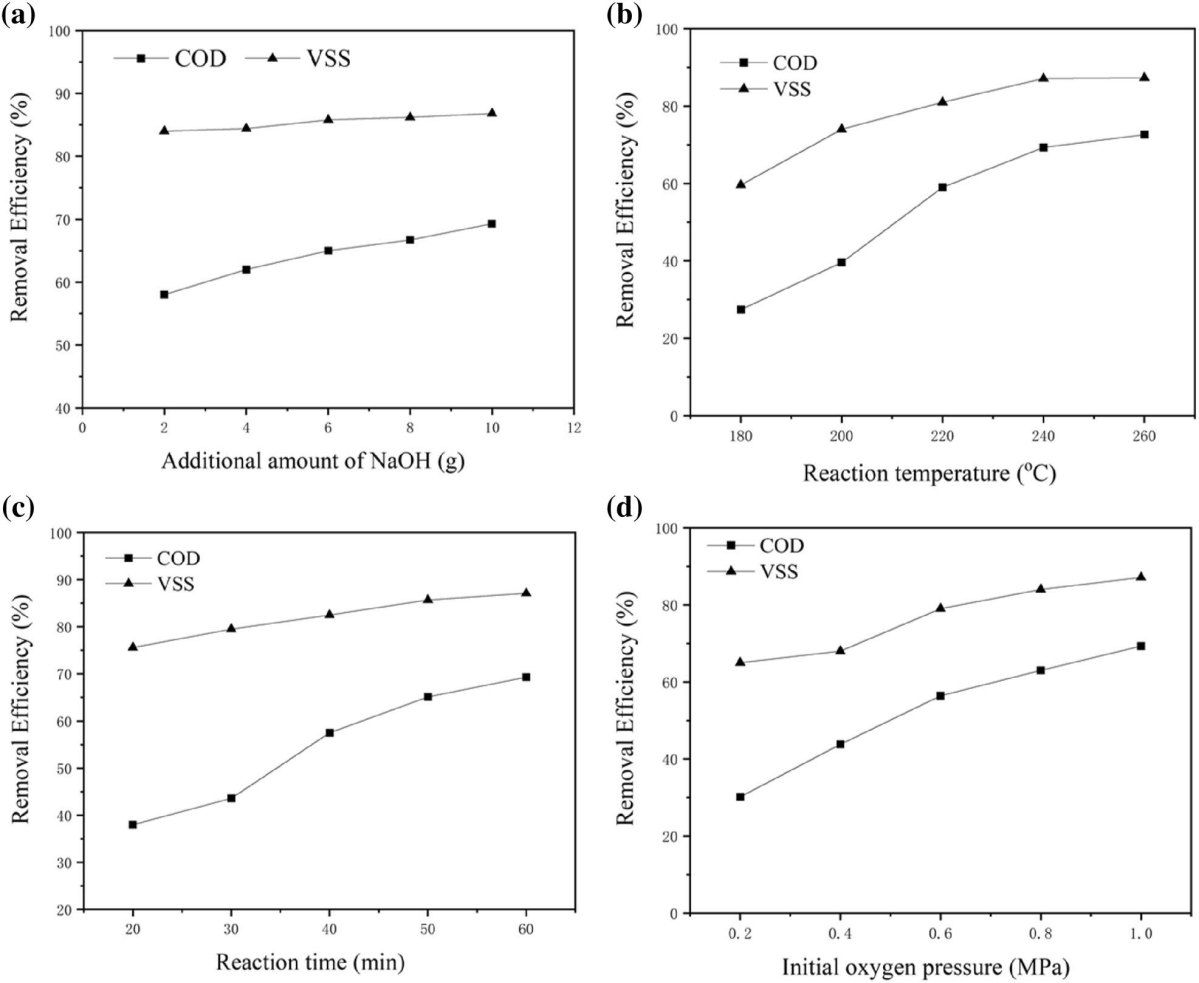
Effect of Cu–Ce/γ-Al_2_O_3_ catalyst on the VSS and COD removal efficiencies.

We examined the performance of the prepared catalyst in catalytic wet oxidation of pharmaceutical sludge^30^. It was found that the highest removal rates for VSS (87.3%) and COD (72.6%) were achieved at 260 °C over 60 min with an oxygen pressure of 1.0 MPa and 10 g·L^−1^ of the catalyst. As shown in Fig. 6a, the COD removal efficiency increased with the rise of catalyst dose. By comparing the results with VSS removal, it was found that the addition of catalyst has little effect on the VSS removal. These results illustrated that the COD and VSS removal may come from different reaction pathways. The effect of the reaction temperature on catalytic wet oxidation is also highly significant, (see Fig. 6b). When the temperature was increased from 180 to 240 °C, the removal rates of COD and VSS increased significantly, while no clear improvement was observed from 240 to 260 °C. This is most likely due the incomplete degradation of COD, because even the organic pollutants in the sludge were almost completely oxidized. In addition, the acetic acid produced in the wet oxidation process is not easily oxidized under hydrothermal conditions. The effects of reaction time can be seen in Fig. 6c. As shown in Fig. 6c, the removal rate gradually increased along with the time. However, in the initial stage, the COD removal rate did not increase, whereas after 20–30 min, the COD removal rate increased strongly. The reason may be that solid-phase organic matter transferred into the liquid phase with the reaction proceeding, then the COD removal rate greatly improved. As the concentration of the organic intermediates in the liquid decreased, the COD removal rate decreased. Figure 6d shows the effect of initial oxygen pressure on the removal efficiency. As shown in Fig. 6d, the initial oxygen pressure strongly affects the COD and VSS removal rates. The removal rate of COD increased strongly as the pressure was increased from 0.2 to 0.6 MPa. These results mean that high oxygen pressure can effectively accelerate the oxidation reaction rate and eliminate organic compounds, resulting in the achievement of high COD and VSS removal^31^.

### Proposed reaction mechanism

**Figure 7:**
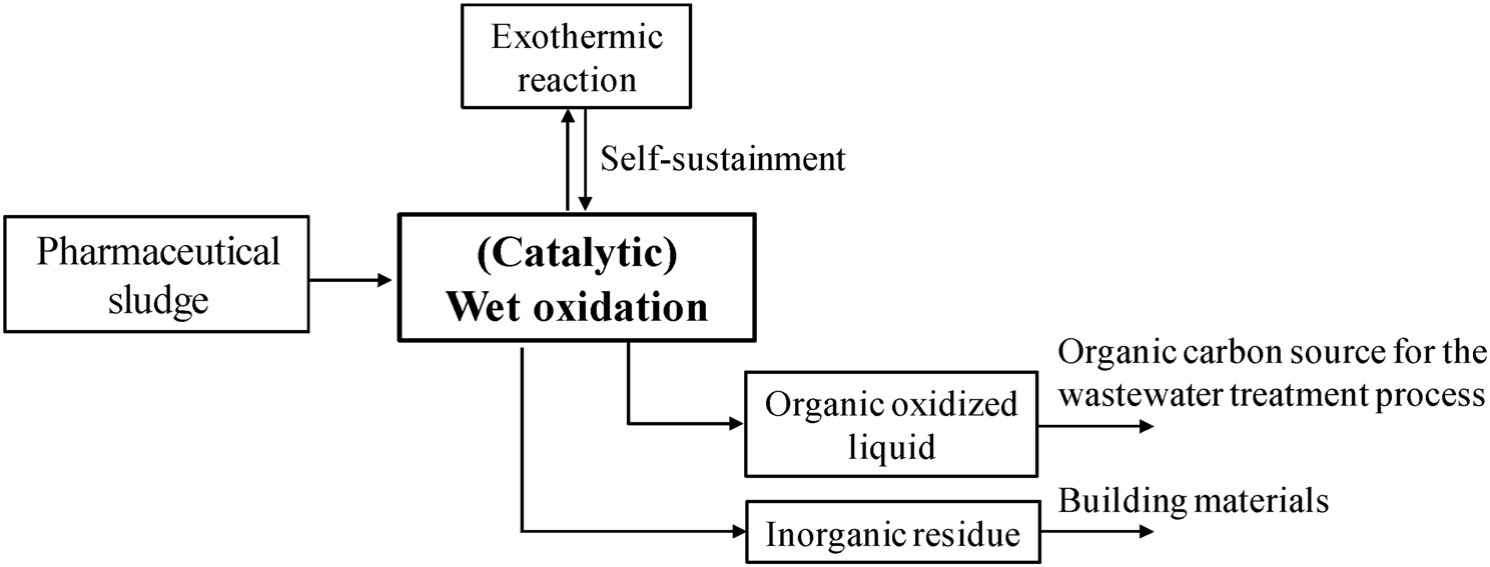
Proposed wet oxidation process of pharmaceutical sludge.

Normally, the wet oxidation reaction is considered a free radical oxidation reaction. To date, relevant mechanistic studies on the wet oxidation of sludge have been quite rare. Therefore, the reaction mechanism of wet oxidation of some organic compounds is still unclear. Because large amounts of intermediates are produced in the wet oxidation process, the reaction mechanism of wet oxidation is always very complicated. For the wet oxidation of pharmaceutical sludge, we proposed the oxidation process shown in Fig. 7. In the first step, the thermal hydrolysis of sludge occurs, producing mixed liquor with soluble organic compounds and inorganic solids. As a result, the VSS removal rate was very high even for a very short reaction time and quite low temperatures. In the second step, the soluble organic compounds reacted with free radical species formed with oxygen gas under hydrothermal conditions and produced carboxylic acids such as acetic acid. The produced low-molecular-weight carboxylic acids, including acetic acid, may be beneficial for further commercial development and can be utilized as organic carbon sources in the wastewater treatment process. With a removal efficiency of VSS above 90%, the residues of the sludge, mainly inorganic solids, can be easily utilized for the production of building materials. From the point of view of energy consumption, exothermic reactions can maintain itself owing to the oxidation of the pollutants. Therefore, catalytic wet oxidation can be regarded as an ideal method for the treatment of pharmaceutical sludge.

## Conclusions

In this work, wet oxidation and catalytic wet oxidation of pharmaceutical sludge using several different catalysts were investigated. The results indicate that wet oxidation is a promising method for the highly efficient degradation of pharmaceutical sludge. Under optimal conditions, the highest removal efficiencies of VSS 86.8% and COD 62.5% were achieved at 260 °C for 60 min with an initial oxygen pressure of 1.0 MPa and initial COD 15,000 mg·L^−1^. Homogeneous catalysts such as Ni^2+^, Fe^3+^, Cu^2+^ and Ce^3+^ ions can significantly enhance the wet oxidation efficiency. By comparison, NaOH can accelerate the wet oxidation of the sludge, particularly with regard to the VSS removal efficiency. The highest VSS removal efficiency of 95.2% was obtained at 260 °C for 60 min with an initial oxygen pressure of 1.0 MPa and 10 g·L^−1^ of NaOH. However, the addition of NaOH inhibited the removal of COD. The Cu–Ce/γ-Al_2_O_3_ catalyst was prepared by a typical wet impregnation procedure. Using this catalyst, the highest removal rates of VSS 87.3% and COD 72.6% were achieved at 260 °C over 60 min with an initial oxygen pressure of 1.0 MPa and 10 g·L^−1^ of the catalyst. In the wet oxidation of the sludge, a thermal hydrolysis process occurred first, producing mixed liquor with soluble organic compounds and inorganic solids. Subsequently, the soluble organic compounds reacted with free radical species formed with oxygen gas under hydrothermal conditions and produced carboxylic acids such as acetic acid. The produced low-molecular-weight carboxylic acids have potential for further commercial utilization as organic carbon sources in wastewater treatment processes. The residues, mainly inorganic solids, can be fed into the klinker furnace of cement production for disposal, or be utilized as construction materials as an additive. From the point of view of energy consumption, exothermic reactions can maintain the reaction owing to the oxidation of pollutants. Therefore, wet oxidation can be regarded as an ideal method for the volume reduction and resource utilization of pharmaceutical sludge.

## Data Availability

The datasets used and analyzed during the current study are available from the corresponding author on reasonable request.
